# An Efficient Method for Systems of Variable Coefficient Coupled Burgers' Equation with Time-Fractional Derivative

**DOI:** 10.1155/2013/687695

**Published:** 2013-10-10

**Authors:** Hossein Aminikhah, Nasrin Malekzadeh

**Affiliations:** Department of Applied Mathematics, School of Mathematical Sciences, University of Guilan, P.O. Box 1914, Rasht 41938, Iran

## Abstract

A new homotopy perturbation method (NHPM) is applied to system of variable coefficient coupled Burgers' equation with time-fractional derivative. The fractional derivatives are described in the Caputo fractional derivative sense. The concept of new algorithm is introduced briefly, and NHPM is examined for two systems of nonlinear Burgers' equation. In this approach, the solution is considered as a power series expansion that converges rapidly to the nonlinear problem. The new approximate analytical procedure depends on two iteratives. The modified algorithm provides approximate solutions in the form of convergent series with easily computable components. Results indicate that the introduced method is promising for solving other types of systems of nonlinear fractional-order partial differential equations.

## 1. Introduction

In recent years, the differential equations of fractional order have been the focus of many studies due to their frequent appearance in various applications in fluid mechanics, medical sciences, biological research, as well as various chemical, biochemical, and physical fields, viscoelasticity, biology, physics, and engineering. Consequently, considerable attention has been given to the solutions of fractional differential equations and integral equations of physical interest [1–4]. Various powerful methods have been presented so far such as homotopy perturbation method [5, 6], variational iteration method [7], differential transform method [8], homotopy analysis method [9], and Adomian decomposition method [10, 11] for solving different kinds of fractional partial differential equations. In this paper, we construct the solution of a system of variable coefficient coupled Burgers' equation with time-fractional derivative by extending the idea of [12, 13]. A new version of homotopy perturbation method is proposed, which we called it NHPM and then applied it to the nonlinear systems of variable coefficient coupled Burgers' equation with time-fractional derivative that can be written as the following basic form:

(1) $$\begin{array}{c} \frac{\partial^{\alpha }u}{\partial t^{\alpha }}=r_{1}\left(t\right)\frac{\partial^{2}u}{\partial x^{2}}+s_{1}\left(t\right)u\frac{\partial u}{\partial x}+p_{1}\left(t\right)\frac{\partial \left(uv\right)}{\partial x},\\ \frac{\partial^{\alpha }v}{\partial t^{\alpha }}=r_{2}\left(t\right)\frac{\partial^{2}v}{\partial x^{2}}+s_{2}\left(t\right)v\frac{\partial v}{\partial x}+p_{2}\left(t\right)\frac{\partial \left(uv\right)}{\partial x}, \end{array}$$

subject to the initial condition

(2) $$\begin{array}{c} u\left(x,0\right)=f\left(x\right),\;\;v\left(x,0\right)=g\left(x\right), \end{array}$$

where the subscripts *r*_1_(*t*), *r*_2_(*t*), *s*_1_(*t*), *s*_2_(*t*), *p*_1_(*t*), and *p*_2_(*t*) are arbitrary smooth functions of *t*.

The paper is organized as follows. In Section 2, we begin with an introduction to some necessary definitions of fractional calculus theory. In Section 3, we illustrated a basic idea of the new method. In Section 4, the uses of the new method for solving nonlinear variable coefficient coupled Burgers' equation are presented. Two examples are solved by the proposed method in this section. Conclusion will appear in Section 5.

## 2. Fractional Calculus

We give some basic definitions and properties of the fractional calculus theory used in this work. Some of these are Riemann-Liouville, Grunwald-Letnikov, Caputo, and generalized functions approach. The most commonly used definitions are the Riemann-Liouville and Caputo derivatives.

**Definition 1**

The Riemann-Liouville fractional integral operator *J* ^*μ*^ of order *μ* on the usual Lebesgue space *L*_1_[*a*, *b*] is given by

(3) $$\begin{array}{c} J^{\mu }f\left(x\right)=\frac{1}{\Gamma \left(\mu \right)}\overset{x}{\underset{0}{\int }}{\left(x-t\right)}^{\mu -1}f\left(t\right)dt,\;\mu >0,\\ J^{0}f\left(x\right)=f\left(x\right). \end{array}$$

It has the following properties:

- *J* ^*μ*^ exists for any *x* ∈ [*a*, *b*],
- *J* ^*μ*^ *J* ^*β*^ = *J* ^*μ*+*β*^,
- *J* ^*μ*^ *J* ^*β*^ = *J* ^*β*^ *J* ^*μ*^,
- *J* ^*μ*^ *J* ^*β*^ *f*(*x*) = *J* ^*β*^ *J* ^*μ*^ *f*(*x*),
- *J* ^*μ*^(*x* − *a*)^*γ*^ = (Γ(*γ* + 1)/Γ(*μ* + *γ* + 1))(*x* − *a*)^*μ*+*γ*^,

where *f* ∈ *L*_1_[*a*, *b*], *μ*, *β* ≥ 0, and *γ* > −1.

The Riemann-Liouville fractional derivative is mostly used by mathematicians, but this approach is not suitable for physical problems of the real world since it requires the definition of fractional order initial conditions which have no physically meaningful explanation yet. Caputo introduced an alternative definition, which has the advantage of defining integer-order initial conditions for fractional order differential equations.

**Definition 2**

The Caputo definition of fractal derivative operator is given by

(4) Dμf(x)=Jm−μDnf(x)=1Γ(m−μ)∫0t(x−τ)m−μ−1fm(τ)dτ,

where *m* − 1 < *μ* ≤ *m*, *m* ∈ *N*, *x* > 0.

**Lemma 3**

If *m* − 1 < *μ* ≤ *m*, *m* ∈ *ℕ*, and *f* ∈ *L*_1_[*a*, *b*], then

(5) DμJμf(x)=f(x),JμDμf(x)=f(x)−∑k=0m−1fk(0+)(x−a)kk!,x>0.

The Caputo fractional derivative is considered here because it allows traditional initial and boundary conditions to be included in the formulation of the problem. In this paper, we have considered some systems of linear and nonlinear FPDEs, where fractional derivatives are taken in Caputo sense as follows.

**Definition 4**

For *n* to be the smallest integer that exceeds *α*, the Caputo time-fractional derivative operator of *α* > 0 is defined as

(6) Dtαu(x,t)=∂αu(x,t)∂tα={1Γ(n−α) ×∫0t(x−τ)n−α−1∂nu(x,τ)∂τndτ,for  n−1<α<n∂nu(x,τ)∂τn,for  α=n∈N.

## 3. Analysis of New Homotopy Perturbation Method 

Let us consider the system of nonlinear fractional differential equations

(7) Dtαui(x,t)=Ai(ui)+fi(t,x),x,t∈Ω, i=1,2,…,n,

with the following initial conditions:

(8) $$\begin{array}{c} u_{i}\left(x,0\right)=\alpha_{i},\;i=1,2,\ldots ,n, \end{array}$$

where *A*_*i*_ are the operators, *f*_*i*_ are known functions, and *u*_*i*_ are sought functions. Assume that operators *A*_*i*_ can be written as

(9) $$\begin{array}{c} A_{i}\left(u_{i}\right)=L_{i}\left(u_{i}\right)+N_{i}\left(u_{i}\right), \end{array}$$

where *L*_*i*_ are the linear operators and *N*_*i*_ are the nonlinear operators. Hence, (7) can be rewritten as follows:

(10) $$\begin{array}{c} D_{t}^{\alpha }u_{i}\left(x,t\right)=L_{i}\left(u_{i}\right)+N_{i}\left(u_{i}\right)+f_{i}\left(x,t\right). \end{array}$$

For solving system (7) by NHPM, we construct the following homotopy:

(11) H(Ui;p)=(1−p)(DtαUi(x,t)−ui,0) +p(Dtαui(x,t)−Li(Ui)−Ni(Ui)−fi(t,x))=0,

where *p* ∈ [0,1] is an embedding or homotopy parameter, *H*(*t*, *x*; *p*) : *Ω* × [0,1] → *R*, and *u*_*i*,0_ are the initial approximation of solution of the problem in (10).

Clearly, the homotopy equations *H*(*U*_*i*_ : 0) = 0 and *H*_*i*_(*U*_*i*_ : 1) = 0 are equivalent to the equations *D*_*t*_^*α*^ *U*_*i*_(*x*, *t*) − *u*_*i*,0_ = 0 and *D*_*t*_^*α*^ *U*_*i*_(*x*, *t*) − *L*_*i*_(*U*_*i*_) + *N*_*i*_(*U*_*i*_) + *f*_*i*_(*t*, *x*) = 0, respectively. Thus, a monotonous change of parameter *p* from zero to one corresponds to a continuous change of the trivial problem *D*_*t*_^*α*^ *U*_*i*_(*x*, *t*) − *u*_*i*,0_ = 0 to the original problem. Next, we assume that the solution of equation *H*(*U*_*i*_, *p*) can be written as a power series in embedding parameter *p* as follows:

(12) $$\begin{array}{c} U_{i}=U_{i,0}+pU_{i,1},\;i=1,2,\ldots ,n. \end{array}$$

Now, let us write (12) in the following form:

(13) $$\begin{array}{c} D_{t}^{\alpha }U_{i}\left(x,t\right)=u_{i,0}+p\left(L_{i}\left(U_{i}\right)+N_{i}\left(U_{i}\right)+f_{i}\left(t,x\right)\right). \end{array}$$

Applying the inverse operator, *J*_*t*_^*α*^, which is the Riemann-Liouville fractional integral of order *α* ≥ 0, on both sides of (13), we have

(14) Ui(x,t)=Ui(x,0)+Jtαui,0+pJtα(Li(Ui)+Ni(Ui)+fi(t,x)).

Suppose that the initial approximation of (10) has the form

(15) ui,0(x,t)=∑n=0∞ai,n(x)pn(t), i=1,2,…,n,

where *a*_*i*,*n*_(*x*), *n* = 0,1, 2,… are unknown coefficients and *p*_*n*_(*t*), *n* = 0,1, 2,… are specific functions on the problem. By substituting (12) and (15) into (14), we get

(16) Ui,0+pUi,1=Ui(x,0)+Jtα(∑n=0∞ai,n(x)pn(t))  +pJtα(Li(Ui,0+pUi,1)+Ni(Ui,0+pUi,1)+fi(t,x)).

Equating the coefficients of like powers of *p*, we get the following set of equations:

(17) coefficient  of  p0:Ui,0(x,t)=Ui(x,0)+Jtα(∑n=0∞ai,n(x)pn(t)),coefficient  of  p1:Ui,1(x,t)=Jtα(Li(Ui,0)+Ni(Ui,0)+fi(t,x)).

Now, we solve these equations in such a way that *U*_*i*,1_(*x*, *t*) = 0. Therefore, the approximate solution may be obtained as

(18) ui(x,t)=Ui,0(x,t)=Ui(x,0)+Jtα(∑n=0∞ai,n(x)pn(t)).

## 4. Examples

In this section, to illustrate the method and to show the ability of the method, two examples are presented.

**Example 1**

Consider the following variable coefficient coupled Burgers' equation:

(19) Dtαu(x,t)=t1−t∂2u(x,t)∂x2−u(x,t)∂u(x,t)∂x  +1+t1−t∂(u(x,t)v(x,t))∂x,Dtαv(x,t)=t1+t∂2v(x,t)∂x2+v(x,t)∂v(x,t)∂x  −1−t1+t∂(u(x,t)v(x,t))∂x,t≠−1,1,

subject to the initial condition

(20) $$\begin{array}{c} u\left(x,0\right)=v\left(x,0\right)=x. \end{array}$$

The exact solutions of (19) for the special case *α* = 1 are *u*(*x*, *t*) = *x*/(1 − *t*) and *v*(*x*, *t*) = *x*/(1 + *t*).

To obtain the solution of (19) by NHPM, we construct the following homotopy:

(21) (1−p)(DtαU(x,t)−u0(x,t)) +p(DtαU(x,t)−t1−t∂2U(x,t)∂x2      +U(x,t)∂U(x,t)∂x−1+t1−t∂(U(x,t)V(x,t))∂x)=0,(1−p)(DtαV(x,t)−v0(x,t)) +p(DtαV(x,t)−t1+t∂2V(x,t)∂x2       −V(x,t)∂V(x,t)∂x+1−t1+t∂(U(x,t)V(x,t))∂x)=0.

Applying the inverse operator *J*_*t*_^*α*^ of *D*_*t*_^*α*^ on both sides of the above equation, we obtain

(22) U(x,t)=U(x,0)+Jtαu0(x,t)−pJtα(u0(x,t)−t1−t∂2U(x,t)∂x2               +U(x,t)∂U(x,t)∂x               −1+t1−t∂(U(x,t)V(x,t))∂x),V(x,t)=V(x,0)+Jtαv0(x,t)−pJtα(v0(x,t)−t1+t∂2V(x,t)∂x2               −V(x,t)∂V(x,t)∂x               +1−t1+t∂(U(x,t)V(x,t))∂x).

For solving system (22), by new homotopy perturbation method, we use the Taylor series of

(23) $$\begin{array}{c} \frac{1}{1-t}=\overset{\infty }{\underset{n=0}{\sum }}t^{n},\\ \frac{1}{1+t}=\overset{\infty }{\underset{n=0}{\sum }}{\left(-1\right)}^{n}t^{n}. \end{array}$$

The solution of (19) has the following form:

(24) $$\begin{array}{c} U\left(x,t\right)=U_{0}\left(x,t\right)+pU_{1}\left(x,t\right),\\ V\left(x,t\right)=V_{0}\left(x,t\right)+pV_{1}\left(x,t\right). \end{array}$$

Substituting (23) and (24) in (22) and equating the coefficients of like powers of *p*, we get the following set of equations:

(25) U0(x,t)=U(x,0)+Jtαu0(x,t),V0(x,t)=V(x,0)+Jtαv0(x,t),U1(x,t)=Jtα(−u0(x,t)+t∑n=0∞tn∂2U0(x,t)∂x2    −U0(x,t)∂U0(x,t)∂x    +(1+t)∑n=0∞tn∂(U0(x,t)V0(x,t))∂x),V1(x,t)=Jtα(−v0(x,t)+t∑n=0∞(−1)ntn∂2V0(x,t)∂x2    +V0(x,t)∂V0(x,t)∂x−(1−t)    ×∑n=0∞(−1)ntn∂(U0(x,t)V0(x,t))∂x).

Assuming *u*_0_(*x*, *t*) = ∑_*n*=0_^*∞*^ *a*_*n*_(*x*)*p*_*n*_(*t*), *v*_0_(*x*, *t*) = ∑_*n*=0_^*∞*^ *b*_*n*_(*x*)*p*_*n*_(*t*), *p*_*n*_(*t*) = *t* ^*nα*^, *U*(*x*, 0) = *u*(*x*, 0), and *V*(*x*, 0) = *v*(*x*, 0) and solving the above equation for *U*_1_(*x*, *t*) and *V*_1_(*x*, *t*) lead to the result

(26) U1(x,t)=(x−a0(x))tαΓ(α+1) +(−a1(x)+b0(x)+xdb0(x)dx+4x) ×Γ(α+1)t2αΓ(2α+1) +(−a2(x)−a0(x)da0(x)dx+d2a0(x)dx2    +12b1(x)+12xdb1(x)dx+da0(x)dxb0(x)    +a0(x)db0(x)dx+2a0(x)+2xda0(x)dx    +2b0(x)+2x(db0(x)dx)+4x) ×Γ(2α+1)t3αΓ(3α+1) +(4x+2a0(x)+⋯+2xdb0(x)dx) ×Γ(3α+1)t4αΓ(4α+1)+⋯,V1(x,t)=(−x−b0(x))tαΓ(α+1) +(−b1(x)−a0(x)−xda0(x)dx+4x) ×Γ(α+1)t2αΓ(2α+1) +(−b2(x)+b0(x)db0(x)dx−d2a0(x)dx2     −12a1(x)−12xda1(x)dx−da0(x)dxb0(x)     −a0(x)db0(x)dx+2a0(x)+2x(da0(x)dx)     +2b0(x)+2x(db0(x)dx)−4x) ×Γ(2α+1)t3αΓ(3α+1) +(4x−2a0(x)+⋯−2x(db0(x)dx)) ×Γ(3α+1)t4αΓ(4α+1)+⋯.

Vanishing *U*_1_(*x*, *t*) and *V*_1_(*x*, *t*) lets the coefficients *a*_*i*_, *b*_*i*_, *i* = 0,1, 2,… have the following values:

(27) $$\begin{array}{c} a_{0}\left(x\right)=x,\;\;a_{1}\left(x\right)=2x,\;\;a_{2}\left(x\right)=3x,\\ a_{3}\left(x\right)=4x,\;\;a_{4}\left(x\right)=5x,\;\;a_{5}\left(x\right)=6x,\ldots ,\\ b_{0}\left(x\right)=-x,\;\;b_{1}\left(x\right)=2x,\;\;b_{2}\left(x\right)=-3x,\\ b_{3}\left(x\right)=4x,\;\;b_{4}\left(x\right)=-5x,\;\;b_{5}\left(x\right)=6x. \end{array}$$

Therefore, we obtain the solutions of (19) as

(28) u(x,t)=x+xtαΓ(α+1)+2xΓ(α+1)t2αΓ(2α+1) +3xΓ(2α+1)t3αΓ(3α+1)+4xΓ(3α+1)t4αΓ(4α+1)+⋯=x(1+∑n=1∞nΓ((n−1)α+1)tnαΓ(nα+1)),v(x,t)=x−xtαΓ(α+1)+2xΓ(α+1)t2αΓ(2α+1) −3xΓ(2α+1)t3αΓ(3α+1)+4xΓ(3α+1)t4αΓ(4α+1)−⋯=x(1+∑n=1∞n(−1)nΓ((n−1)α+1)tnαΓ(nα+1)).

If we put *α* → 1 in (28) or solve (19) with *α* = 1, we obtain the exact solution

(29) u(x,t)=x(1+t+t2+t3+⋯)=x1−t,v(x,t)=x(1−t+t2−t3+⋯)=x1+t.

**Example 2**

Consider the following variable coefficient coupled Burgers' equation:

(30) Dtαu(x,t)=−∂2u(x,t)∂x2+2e2tu(x,t)∂u(x,t)∂x −sin(2t)∂(u(x,t)v(x,t))∂x,Dtαv(x,t)=∂2v(x,t)∂x2−2e−2tcos⁡(2t)v(x,t)∂v(x,t)∂x +cos⁡(2t)∂(u(x,t)v(x,t))∂x,

subject to the initial condition

(31) $$\begin{array}{c} u\left(x,0\right)=v\left(x,0\right)=e^{x}. \end{array}$$

The exact solution for *α* = 1 is *u*(*x*, *t*) = *e* ^*x*−*t*^and *v*(*x*, *t*) = *e* ^*x*+*t*^.

To obtain the solution of (30) by NHPM, we construct the following homotopy:

(32) (1−p)(DtαU(x,t)−u0(x,t))  +p(DtαU(x,t)+∂2U(x,t)∂x2       −2e2tsin(2t)U(x,t)∂U(x,t)∂x       +sin(2t)∂(U(x,t)V(x,t))∂x)=0,(1−p)(DtαV(x,t)−v0(x,t))  +p(DtαV(x,t)−∂2V(x,t)∂x2       +2e−2tcos⁡(2t)V(x,t)∂V(x,t)∂x       −cos⁡(2t)∂(U(x,t)V(x,t))∂x)=0.

Applying the inverse operator *J*_*t*_^*α*^ of *D*_*t*_^*α*^ on both sides of the above equation, we obtain

(33) U(x,t)=U(x,0)+Jtαu0(x,t) −pJtα(u0(x,t)+∂2U(x,t)∂x2       −2e2tsin(2t)U(x,t)∂U(x,t)∂x       +sin(2t)∂(U(x,t)V(x,t))∂x),V(x,t)=V(x,0)+Jtαv0(x,t) −pJtα(v0(x,t)−∂2V(x,t)∂x2       +2e−2tcos⁡(2t)V(x,t)∂V(x,t)∂x       −cos⁡(2t)∂(U(x,t)V(x,t))∂x).

For solving system (33), by new homotopy perturbation method, we use the Taylor series of

(34) $$\begin{array}{c} \sin\left(2t\right)=\overset{\infty }{\underset{n=0}{\sum }}{\left(-1\right)}^{n}\frac{{\left(2t\right)}^{2n+1}}{\left(2n+1\right)!},\\ \cos\left(2t\right)=\overset{\infty }{\underset{n=0}{\sum }}{\left(-1\right)}^{n}\frac{{\left(2t\right)}^{2n}}{\left(2n\right)!},\\ \exp\left(2t\right)=\overset{\infty }{\underset{n=0}{\sum }}\frac{{\left(2t\right)}^{n}}{n!},\\ \exp\left(-2t\right)=\overset{\infty }{\underset{n=0}{\sum }}{\left(-1\right)}^{n}\frac{{\left(2t\right)}^{n}}{n!}. \end{array}$$

The solution of (30) has the following form:

(35) $$\begin{array}{c} U\left(x,t\right)=U_{0}\left(x,t\right)+pU_{1}\left(x,t\right),\\ V\left(x,t\right)=V_{0}\left(x,t\right)+pV_{1}\left(x,t\right). \end{array}$$

Substituting (34) and (35) in (33) and equating the coefficients of like powers of *p*, we get the following set of equations:

(36) U0(x,t)=U(x,0)+Jtαu0(x,t),V0(x,t)=V(x,0)+Jtαv0(x,t),U1(x,t)=Jtα(−u0(x,t)−∂2U0(x,t)∂x2     +2∑n=0∞(2t)nn!∑n=0∞(−1)n(2t)2n+1(2n+1)!U0(x,t)∂U0(x,t)∂x     −∑n=0∞(−1)n(2t)2n+1(2n+1)!∂U0(x,t)V0(x,t)∂x),V1(x,t)=Jtα(−v0(x,t)+∂2V0(x,t)∂x2     −2∑n=0∞(−1)n(2t)nn!∑n=0∞(−1)n(2t)2n(2n)!     ×V0(x,t)∂V0(x,t)∂x     +∑n=0∞(−1)n(2t)2n(2n)!∂U0(x,t)V0(x,t)∂x).

Assuming *u*_0_(*x*, *t*) = ∑_*n*=0_^*∞*^ *a*_*n*_(*x*)*p*_*n*_(*t*), *v*_0_(*x*, *t*) = ∑_*n*=0_^*∞*^ *b*_*n*_(*x*)*p*_*n*_(*t*), *p*_*n*_(*t*) = *t* ^*nα*^, *U*(*x*, 0) = *u*(*x*, 0), and *V*(*x*, 0) = *v*(*x*, 0) and solving the above equation for *U*_1_(*x*, *t*) and *V*_1_(*x*, *t*) lead to the result

(37) U1(x,t)=(−a0(x)−ex)tαΓ(α+1) +(−a1(x)−d2a0(x)dx2)Γ(α+1)t2αΓ(2α+1) +(−a2(x)−12d2a1(x)dx2−2b0(x)ex    +2a0(x)ex+2exda0(x)dx    −2ex(db0(x)dx)+8e2x)Γ(2α+1)t3αΓ(3α+1) +(−a3(x)−2a0(x)(db0(x)dx)    +⋯+8a0(x)ex) ×Γ(3α+1)t4αΓ(4α+1)+⋯,V1(x,t)=(−b0(x)+ex)tαΓ(α+1) +(−b1(x)−b0(x)ex+a0(x)ex      +exda0(x)dx−exdb0(x)dx      +d2b0(x)dx2+4e2x)Γ(α+1)t2αΓ(2α+1) +(−b2(x)−12exdb1(x)dx    −12b1(x)ex+12a1(x)ex    +a0(x)db0(x)dx+4b0(x)ex) ×Γ(2α+1)t3αΓ(3α+1) +(−b1(x)db0(x)dx−13b2(x)ex    +⋯+13d2b2(x)dx2)Γ(3α+1)t4αΓ(4α+1)+⋯.

Vanishing *U*_1_(*x*, *t*) and *V*_1_(*x*, *t*) lets the coefficients *a*_*i*_, *b*_*i*_, *i* = 0,1, 2,… have the following values:

(38) $$\begin{array}{c} a_{0}\left(x\right)=-e^{x},\;\;a_{1}\left(x\right)=e^{x},\;\;a_{2}\left(x\right)=-\frac{1}{2!}e^{x},\\ a_{3}\left(x\right)=\frac{1}{3!}e^{x},\;\;a_{4}\left(x\right)=-\frac{1}{4!}e^{x},\ldots ,\\ b_{0}\left(x\right)=e^{x},\;\;b_{1}\left(x\right)=e^{x},\;\;b_{2}\left(x\right)=\frac{1}{2!}e^{x},\\ b_{3}\left(x\right)=\frac{1}{3!}e^{x},\;\;b_{4}\left(x\right)=\frac{1}{4!}e^{x},\ldots . \end{array}$$

Therefore, we obtain the solutions of (30) as

(39) u(x,t)=ex−extαΓ(α+1)+exΓ(α+1)t2αΓ(2α+1)  −12!exΓ(2α+1)t3αΓ(3α+1)+13!exΓ(3α+1)t4αΓ(4α+1)−⋯=ex(1+∑n=1∞(−1)nΓ((n−1)α+1)tnα(n−1)!Γ(nα+1)),v(x,t)=ex+extαΓ(α+1)+exΓ(α+1)t2αΓ(2α+1)  +12!exΓ(2α+1)t3αΓ(3α+1)+13!exΓ(3α+1)t4αΓ(4α+1)+⋯=ex(1+∑n=1∞Γ((n−1)α+1)tnα(n−1)!Γ(nα+1)).

If we put *α* → 1 in (39) or solve (30) with *α* = 1, we obtain the exact solution

(40) u(x,t)=ex(1−t+t22!−t33!+···)=ex−t,v(x,t)=ex(1+t+t22!+t33!+···)=ex+t.

## 5. Concluding Remarks

In this paper, we have used a new homotopy perturbation method for solving a system of two nonlinear time-fractional partial differential equations. The NHPM for solving system of variable coefficient coupled Burgers' equation with time-fractional derivative is based on two-component procedure and polynomial initial condition. The Computations finally lead to a set of nonlinear equations with one unspecified value in each equation. This set can be readily solved using Maple, and putting these values into the first approximate solution yields the analytical approximate solution. The present study has confirmed that NHPM offers significant advantages in terms of its straightforward applicability, computational efficiency, and accuracy. Thus, we conclude that the new method can be considered as an efficient method for solving linear and nonlinear problems.
